# Description of a new species of the Neotropical cichlid genus *Gymnogeophagus* Miranda Ribeiro, 1918 (Teleostei: Cichliformes) from the Middle Paraná basin, Misiones, Argentina

**DOI:** 10.1371/journal.pone.0210166

**Published:** 2019-02-13

**Authors:** Felipe Alonso, Guillermo E. Terán, Gastón Aguilera, Oldřich Říčan, Jorge Casciotta, Wilson Sebastián Serra, Adriana Almirón, Mauricio F. Benítez, Ignacio García, Juan Marcos Mirande

**Affiliations:** 1 Instituto de Bio y Geociencias del NOA (IBIGEO-CONICET), CCT-Salta, Rosario de Lerma, Salta, Argentina; 2 Unidad Ejecutora Lillo (CONICET), Fundación Miguel Lillo, San Miguel de Tucumán, Tucumán, Argentina; 3 University of South Bohemia, Faculty of Science, Department of Zoology, České Budějovice, Czech Republic; 4 Universidad Nacional de La Plata, Facultad de Ciencias Naturales y Museo, División Zoología Vertebrados, La Plata, Buenos Aires, Argentina; 5 Comisión de Investigaciones Científicas de la Provincia de Buenos Aires, La Plata, Buenos Aires, Argentina; 6 Sección Ictiología, Dpto. de Zoología, Museo Nacional de Historia Natural, Montevideo, Uruguay; 7 Centro Universitario Regional del Este (CURE) Sede Rocha, Rocha, Uruguay; 8 Sección Zoología de Vertebrados, Facultad de Ciencias de la UdelaR, Montevideo, Uruguay; 9 Instituto de Biología Subtropical (CONICET-UNaM), Posadas, Argentina; 10 Instituto de Limnología "Dr. Raúl A. Ringuelet" (ILPLA-CONICET), La Plata, Buenos Aires, Argentina; Universita degli Studi di Roma La Sapienza, ITALY

## Abstract

*Gymnogeophagus jaryi*, new species, is described from Southern tributaries of the Middle Paraná basin in Misiones. It can be distinguished from all other members of the genus, except from *G*. *australis* and *G*. *caaguazuensis*, by the presence of a hyaline to grey anterior portion of the dorsal fin. *Gymnogeophagus jaryi* differs from *G*. *caaguazuensis* by a longer caudal peduncle, caudal fin not lyrate, central portion of scales on dorsal portion of trunk light iridescent blue and by white spots in soft portion of dorsal fin in adult males, and from *G*. *australis* by the light iridescent blue coloration of central portion of scales on the dorsal portion of trunk and tail, and by the lack of scales on the soft portion of the dorsal fin. Additionally, it can be diagnosed by the following unique combination of characters: 10–11 dorsal-fin branched rays, 27–30 E1 scales, absence of lips thickening, and, in males, by the possession of a hump in adults, caudal fin not lyrate, presence of large white spots forming transversal stripes distally and in anterior area of the dorsal fin’s soft portion, central area of scales on the dorsal portion of the trunk light iridescent blue, lack of scales on the base of the dorsal fin’s soft portion, absence of a conspicuous and oblique dark band from the eye to the anterior border of the head, anterior portion of dorsal fin hyaline to grey, scales of the midlateral spot each bearing a semicircular light blue blotch, head hump starting at the horizontal through the eyes, concave anterior profile in lateral view, base of unpaired fins yellow, and whitish hyaline spots on caudal fin. The new species, based on mtDNA phylogeny, is the sister species of *G*. *caaguazuensis* from the Paraguay basin and is closely related to *G*. *australis*.

## Introduction

Cichlidae is one of the most diverse families of fishes of the world, with almost 600 valid species in the Neotropical region which are included into Cichlinae [1]. Within this subfamily, *Gymnogeophagus* Miranda Ribeiro 1918 includes 18 extant species [2, 3] and one fossil [4], distributed in the La Plata basin, the Laguna dos Patos system, and the río Tramandaí basin, plus a single record of *G*. *balzanii* from the río Guaporé in the Amazon basin [2,5]. *Gymnogeophagus* is diagnosed by the presence of a forward directed spine on top of first dorsal-fin pterygiophore and by the lack of bony supraneurals [6]. The species of *Gymnogeophagus* are informally included in two species groups, the *G*. *gymnogenys* group, characterized by a mouthbrooding reproductive strategy, and the *G*. *rhabdotus* group showing substrate brooding [5].

The highest species richness in the genus is known from the Uruguay River basin, where several new species were described in the last years [2, 5]. However, a new species of the *G*. *rhabdotus* group was described recently from the Middle Paraná basin [3] and Říčan *et al*. [7] highlighted this area as a possible hotspot of fish endemism that also includes several putatively new species of *Gymnogeophagus*. After the revision of specimens from this area in Misiones, we describe herein a new species of the *G*. *gymnogenys* species group, representing the first new species from this species group in this area.

## Results

### *Gymnogeophagus jaryi*, new species

**Fig 1:**
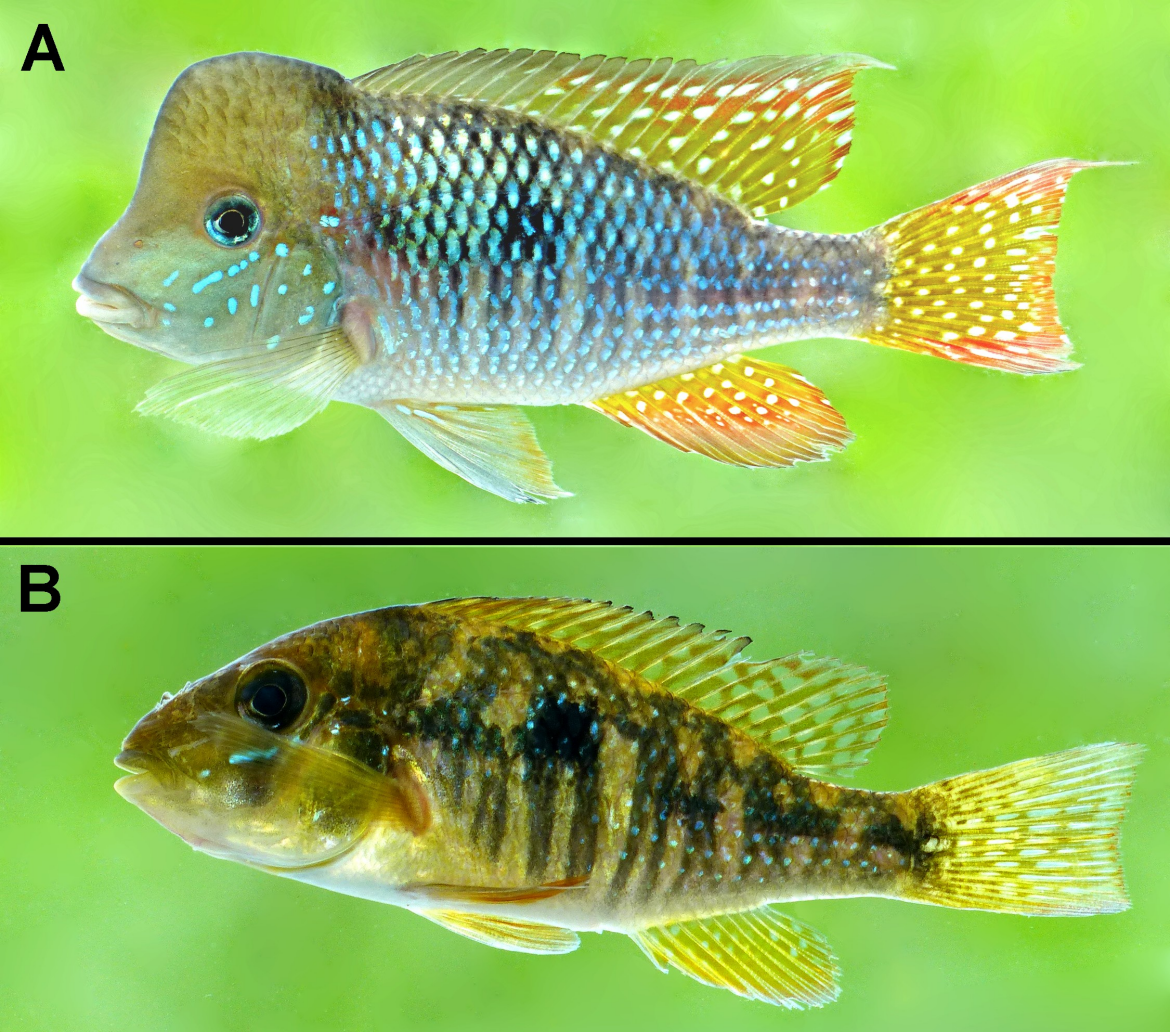
Live specimens of *Gymnogeophagus jaryi* sp. nov. in left lateral view from type locality. From Cuña Pirú stream, Paraná River basin, near Aristóbulo del Valle, Misiones Province, Northeastern Argentina (27°5'16.69"S, 54°57'10.60"W). (A) Holotype, male CI-FML 7463, 113.1 mm SL, (B) Paratype, female CI-FML 7464, 73.3 mm SL.

**Fig 2:**
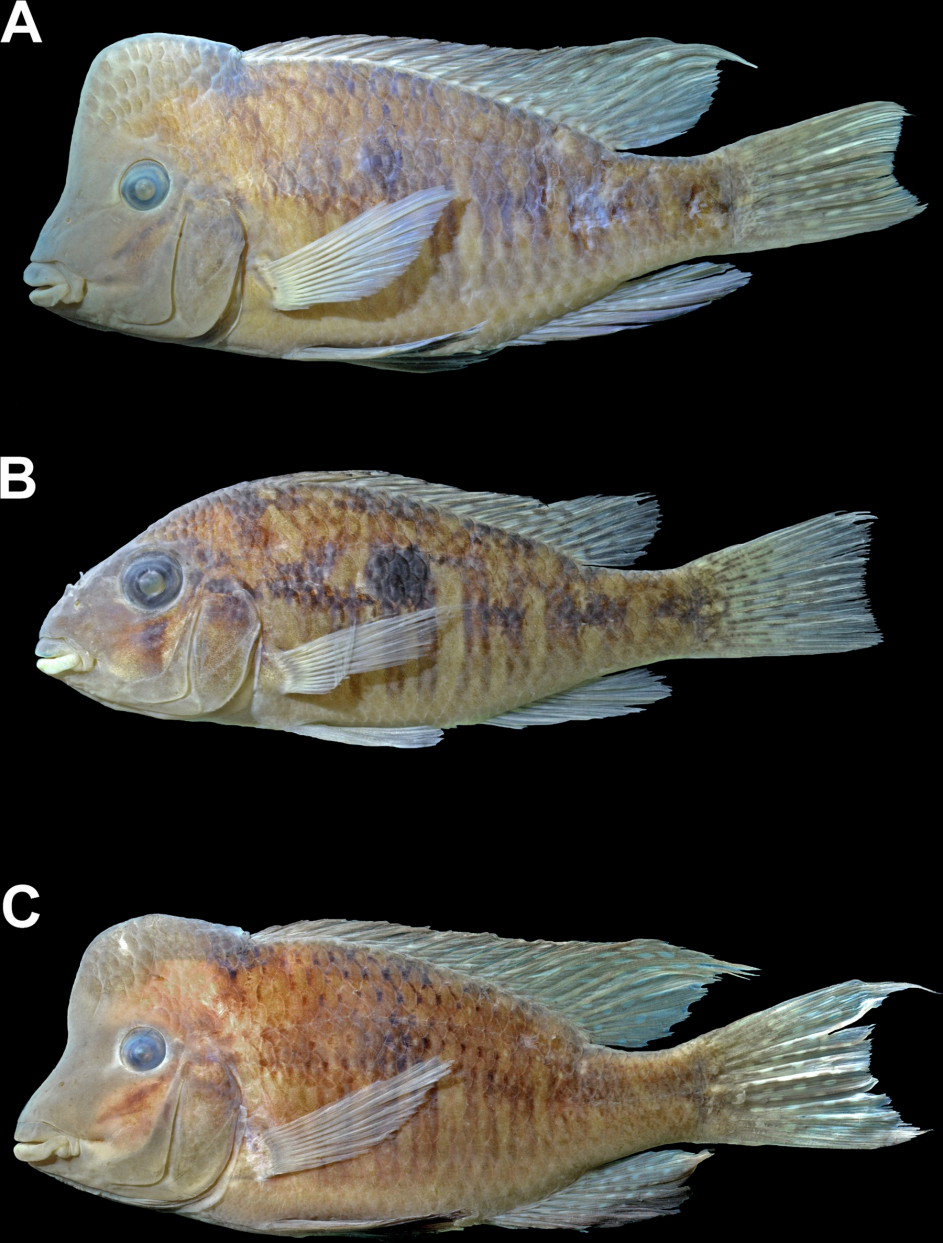
Preserved specimens of *Gymnogeophagus jaryi* sp. nov. in left lateral view from type locality. From Cuña Pirú stream, Paraná River basin, near Aristóbulo del Valle, Misiones Province, Northeastern Argentina (27°5'16.69"S, 54°57'10.60"W). (A) Holotype, male CI-FML 7463, 113.1 mm SL, (B) paratype, female CI-FML 7464, 73.3 mm SL, (C) paratype, male CI-FML 5424, 126.1 mm SL.

**Fig 3:**
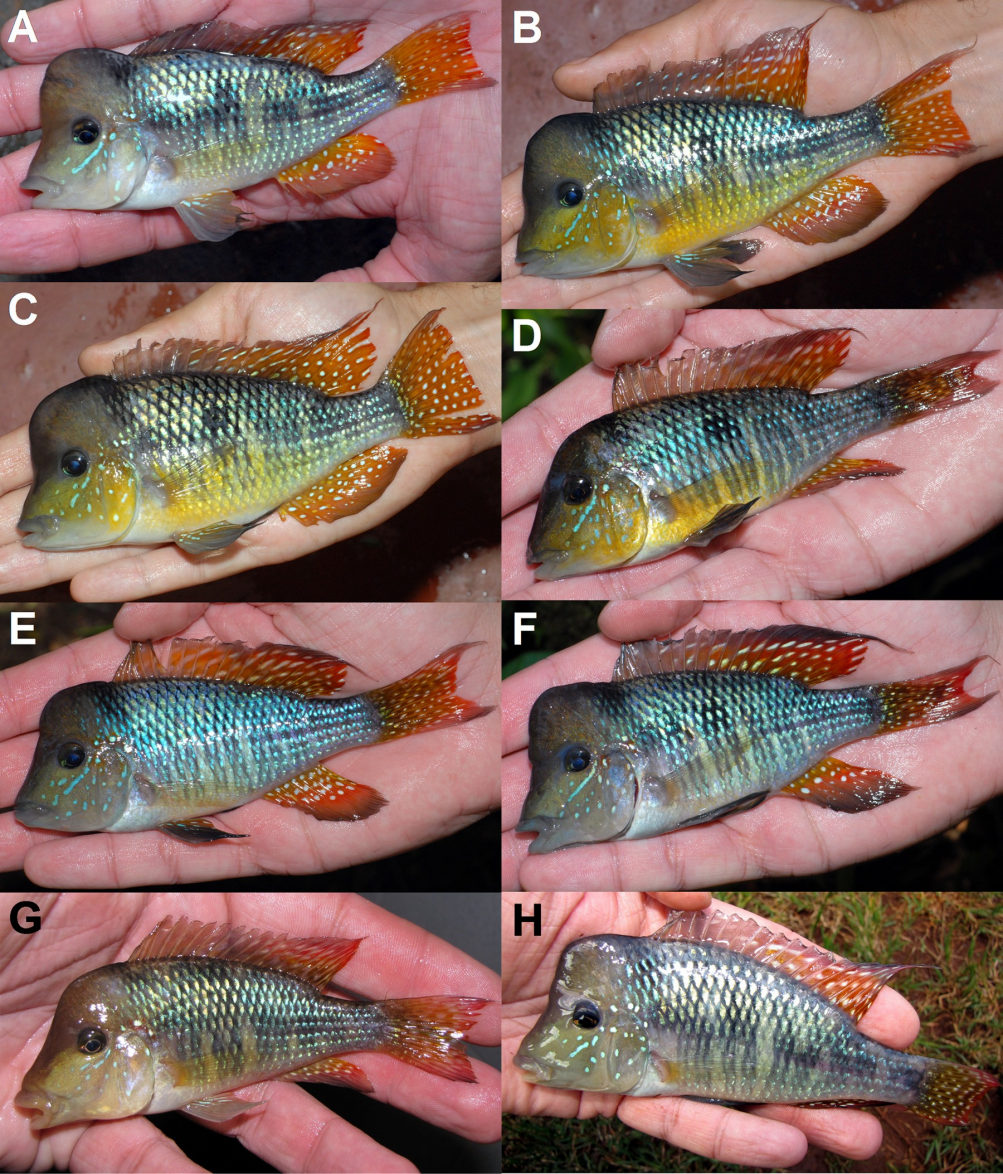
Live specimens of *Gymnogephagus jaryi* sp. nov. in left lateral view, paratype and non-type adult male specimens. (A) MLP 11293, paratype, same locality as holotype (Cuña Pirú); (B,C) MLP 11295, paratype (Cuña Pirú); (D,E,F) MLP 11365, non-type (Ñacanguazú); (G) Paraguay, not preserved (Ype Curu); (H) Paraguay, not preserved (Manduviyú). It needs to be considered that these pictures of the alive specimens have been taken at night and out of water using the camera’s flash light iluminating from the latterals of the fish, which have slightly altered the colors seen. Also, holding them in hand while taking the pictures produced a slight translucent redish aspect to the fins.

urn:lsid:zoobank.org:act:DFC7496E-9587-457A-AA26-B8D31D4973A3 (Figs 1–3), Table 1.

**Table 1:** Frequency distribution of meristic characters.

Dorsal-fin spines	13 (13)*	14 (10)			
Dorsal-fin soft rays	10 (14)	11 (9)*			
Anal-fin rays	III 8 (19)*	III 9 (3)			
Pectoral-fin rays	12 (15)	13 (8)*			
Pelvic-fin rays	I 5 (23)*				
Upper lateral line scales	16 (3)	17 (13)*	18 (6)	19 (1)	
Lower lateral line scales	9 (2)	10 (2)	11 (10)*	12 (7)	13(2)
E1 scales	27 (2)	28 (2)	29 (13)*	30 (6)	

Values based on paratypes and holotype* meristics of *Gymnogeophagus jaryi*, n = 23; (number of specimens).

*Gymnogeophagus* sp. (Fig 12 in [8])

*Gymnogeophagus* sp. 1. (Fig 1 in [7])

### Holotype

CI-FML 7463, male, 113.1 mm SL, Argentina, Misiones, Cuña Pirú stream, 8.5 km from Aristóbulo del Valle on RP7, Paraná River basin (27°5'16.69"S, 54°57'10.60"W), XI-2016, Mirande, Aguilera & Terán.

### Paratypes

All from Argentina, Misiones, Paraná River basin: CI-FML 7464, 2, 66.8–73.3 mm SL, collected with the holotype. IBIGEO-I 462, 3, 66.2–73.3 mm SL, Cuña Pirú stream, same locality as holotype, XII-2004, Mirande & Aguilera. CI-FML 5424, 5, 126.1–71.2 mm SL, 3 c&s, 72.6–104.9 mm SL, Cuña Pirú stream, XII-2004., Mirande & Aguilera MLP 11293, 5, 73.8–106.0 mm SL, same locality as holotype, 5-XII-2007, Říčan, Říčanová, Piálek and Casciotta. CI-FML 7433, 2, 63.2–79.3 mm SL, Aristóbulo del Valle, Cuña Pirú stream, XI-2008, Mirande, Aguilera & Terán. CI-FML 7466, 5, 41.9–67.6 mm SL, Aristóbulo del Valle, Cuña Pirú stream, XII-2004, Mirande & Aguilera. IBIGEO-I 463, 3, 71.6–107.6 mm SL, Tamanduacito stream (27º02'45'' S, 55º00'13'' W), XII-2004, Mirande & Aguilera. MLP 11295, 3, 76.3–132.6 mm SL, Arroyo Cuña Pirú on road 223 near the city of Ruiz de Montoya, 5-XII-2007, Říčan, Říčanová, Piálek and Casciotta. CI-FML 5439, 2, 59.9–83.1 mm SL, Aristóbulo del Valle, Azul stream (aprox. 26°56’22”S; 54°51’22”W), XII-2004, Mirande & Aguilera. CI-FML 5438, 5, 65.5–85.8 mm SL, Aristóbulo del Valle, Tamandua stream (aprox. 26º59'44''S, 54º57'22''W), XII-2004, Mirande & Aguilera. CI-FML 7465, 5, 46.0–61.1 mm SL, collected with the holotype.

### Other non-type material

Argentina, Misiones: MLP 11365, 29, 46.2–116.6mm SL, Arroyo Ñacanguazú (27°07'14.1"S, 55°22'22.1"W), 23-X-2009, Paraná River basin, Říčan, Piálek, Almirón & Casciotta.

### Diagnosis

The number of E1 scales, 27–30 (vs. 23–25), and the possession of a cephalic hump in adult males, distinguishes the new species from all species of the *G*. *rhabdotus* species group (*G*. *rhabdotus*, *G*. *meridionalis*, *G*. *setequedas*, *G*. *che*, *G*. *terrapurpura* and *G*. *taroba*). It is distinguished from all species of the *G*. *gymnogenys* group, except *G*. *caaguazuensis* and *G*. *australis*, by having the anterior portion of the dorsal fin grey to hyaline, in few specimens grey slightly reddish, with no markings (vs. red to yellow with hyaline spots or elongated transversal blotches). It differs from *G*. *caaguazuensis* by a longer caudal peduncle (18.5–22.0 vs. 13.9–17.4, % SL), caudal fin not lyrate (*vs*. lyrate), central portion of scales on dorsal portion of trunk light iridescent blue (*vs*. golden to greenish) and, in adult males, white spots in the soft portion of the dorsal fin, sometimes elongated in the distal portion forming lines (*vs*. with spaced small silvery to bright blue dots in *G*. *caaguazuensis*). It differs from *G*. *australis* by the light iridescent blue coloration of the central portion of scales on the dorsal portion of trunk and tail (*vs*. with golden central portion of scales) and by the lack of scales on dorsal-fin soft portion (vs. present). It is distinguished from *G*. *balzanii* by a lower body depth and less branched dorsal-fin rays (10–11 *vs*. 12–15). It can be further distinguished from *G*. *peliochelynion*, *G*. *labiatus* and *G*. *pseudolabiatus* by the absence of thickening in the lips (*vs*. present); from *G*. *gymnogenys* and *G*. *mekinos* by the absence of a conspicuous and oblique dark band from the anterior margin of eye to the anterior border of head; from *G*. *gymnogenys* also by presence of elongated spots distally in the soft portion of the dorsal fin (vs. large round spots); from *G*. *mekinos* also by dorsal fin coloration (vs. spiny portion without markings, soft portion with only few dots, distally immaculate). Additionally, the new species differs from *G*. *constellatus* by a semicircular light blue spot on each scale of the midlateral spot (*vs*. large white spot) and by spiny posterior portion with short narrow stripes or spots (vs. long wide stripes) and soft portion with dots and lines distally (vs. long wide stripes); from *G*. *tiraparae* by lacking the over-developed head hump (hump starting only at the horizontal plane through the eyes, forming a concave profile of the snout in lateral view vs. hump starting already from the upper lip, forming a convex profile at eyes height) and by a different coloration pattern of the dorsal fin (spiny posterior portion with short narrow stripes or spots and soft portion with dots and lines distally vs. dorsal fin hyaline with two horizontal series of moderately elongated light blue dots between dorsal-fin spines, and a series of light blue stripes between soft rays, and a red ground color between the two series of dots); from *G*. *lipokarenos* by presenting a red distal margin on posterior half of dorsal fin (*vs*. red distal margin along the entire fin), by lower peduncle length 18.5–22.0% of SL (vs. 14.1–17.9% of SL in *G*. *lipokarenos*); from *G*. *missioneiro* by having the base of unpaired fins yellow (*vs*. red) and by the presence of separated dots in both the spiny and soft portions of dorsal fin (vs. long wide stripes in *G*. *missioneiro*); and from *G*. *lacustris* by having lips, branchiostegal membrane and isthmus grey (vs. orange), unpaired fin-bases yellowish (*vs*. light olivaceous to reddish), by hyaline or white spots on caudal fin (*vs*. longitudinal stripes), and by absence of a dark vertical stripe through the eyes (vs. present in *G*. *lacustris*) (Figs 1–3).

### Description

**Fig 4:**
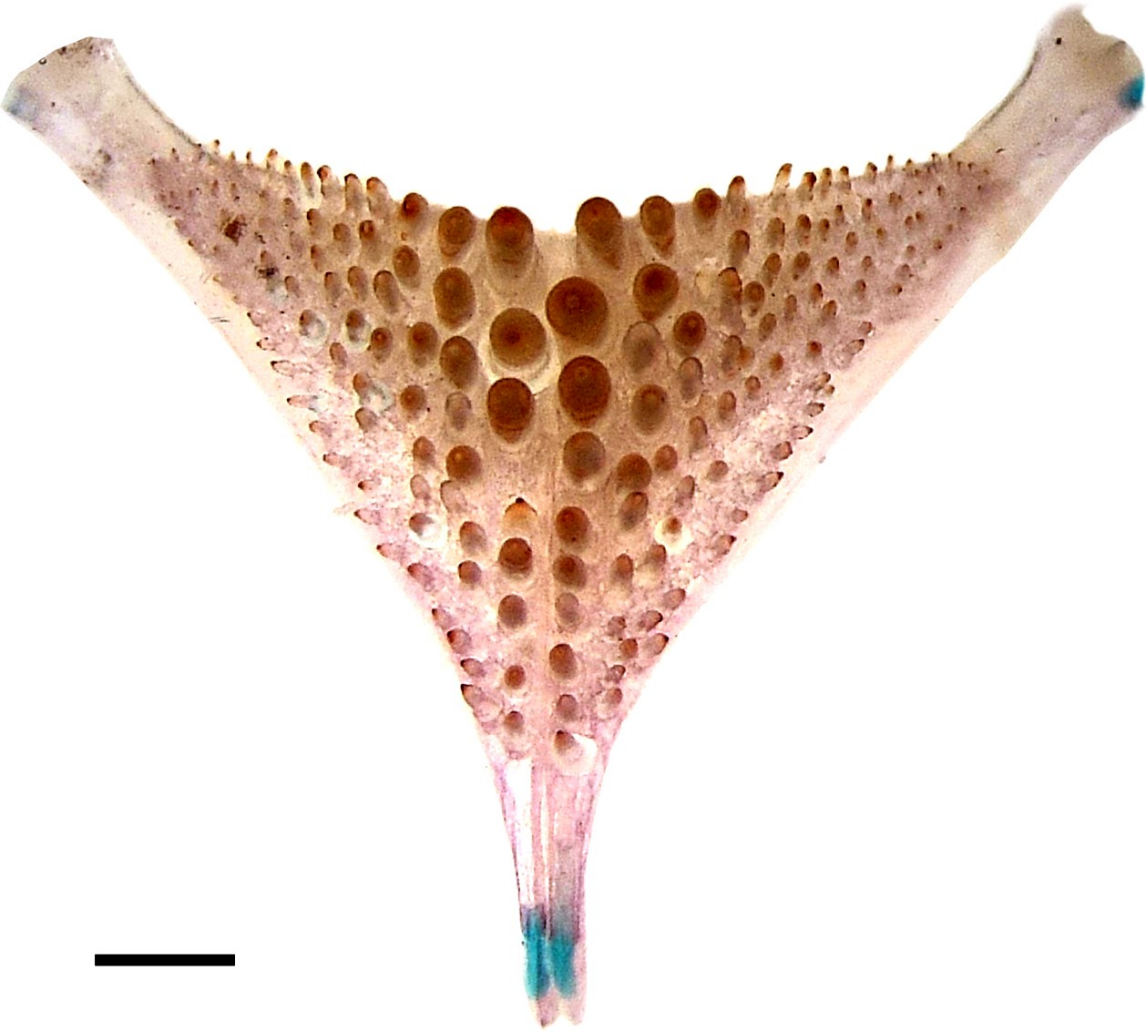
Dorsal view of lower pharyngeal tooth plate. CI-FML 5424. Scale bar = 1 mm.

Meristic and morphometric data summarized in Tables 1 and 2. Holotype and paratypes illustrated in Figs 1–3. Body elongate, laterally compressed. Dorsal profile of head concave between mouth and interorbital area; slightly convex from interorbital region to dorsal fin origin in females and juveniles, while males present a pronounced hump from the nostrils to the dorsal fin origin. Posterior region of hump meeting dorsal fin origin at an obtuse angle. Adipose hump sometimes surrounding origin of dorsal fin in large males. Dorsal fin base convex. Caudal peduncle longer than deep, with dorsal and ventral profiles slightly concave. Snout slightly blunt and rounded dorsally in young; slightly rounded dorsally and slightly rounded to nearly straight ventrally in adults; narrow and anteriorly rounded in dorsal aspect. Eye small, close to dorsal profile of head in juveniles and progressively farther in larger specimens; eye near middle of head length. Posterior tip of maxilla not reaching vertical line across anterior margin of eye. Upper jaw equal or slightly longer than lower jaw. Body scales large and ctenoid, smaller around pectoral fins; scales cycloid in pre-ventral area. Soft portion of dorsal fin of males without scales between contiguous rays. First dorsal-fin spine inserted slightly anterior to vertical line across posterior bony margin of opercle. Posterior distal margin of dorsal fin in young rounded, slightly pointed in adult females and with pointed projections slightly curved ventrally formed by 4th and 5th soft rays in adult males, slightly overpassing caudal-fin base. Anal-fin origin under last dorsal-fin spine or first soft ray; anal fin tip nearly rounded in young and females and pointed in males. Pectoral fin with rounded tip in young and slightly pointed in adults; 3rd ray longest, not reaching vertical crossing anal-fin origin. Pelvic fin pointed, more conspicuously in adult males; 2nd soft ray longest approximately reaching area of anal opening in females and passing anal-fin origin in mature males. Caudal-fin posterior margin concave. Premaxillary teeth slender, conical, with recurved tips. Upper jaw with outer regular row of 20–27 teeth (specimens of 72.2–101.0 mm SL). Medial portion of alveolar premaxillary region with two teeth rows in smaller specimens, three in intermediate sized (74.2 mm SL), and four in largest examined specimen. Dentary teeth strongly recurved posteriorly, forming 4–6 irregular rows in medial portion of dentary; teeth of approximately same size. Outer series with 25–35 teeth (more in larger specimens). Lower limb of first gill arch with 7 gill rakers; upper limb lobed, bearing 4 gill rakers on its margin. Vertebrae 12+16 in three specimens. Lower pharyngeal tooth plate wide (length of bone 89% of its width); dentigerous area of lower pharyngeal tooth plate covering whole occlusion surface; 26–27 teeth on posterior pharyngeal row, 8–10 on median row. Median teeth large, with blunt cusp of molariform aspect. Teeth decreasing laterally, with lateral ones much smaller. Cusps of smaller teeth slightly recurved (Fig 4).

**Table 2:** Morphometrics of *Gymnogeophagus jaryi* sp. nov.

	Holotype	Range	Mean	SD
Standard Length (mm)	113.1	59.9–126.1	79.9.5	18.4
Percents of standard length				
Body depth	42.5	37.3–43.5	39.7	1.9
Head length	35.9	34.3–37.3	35.8	0.8
Dorsal-fin base	54.7	48.9–55.6	52.4	1.7
Pectoral-fin length	30.7	27.9–34.4	31.2	1.4
Caudal peduncle depth	14.0	12.7–14.7	13.5	0.5
Caudal peduncle length	19.5	18.5–22.0	19.8	1.9
Percents of head length				
Horizontal eye diameter	20.7	19.4–29.9	24.6	2.9
Interorbital width	28.7	24.9–30.4	27.6	1.6
Upper jaw length	35.8	30.6–35.8	32.9	1.3
Pre-orbital width	33.0	25.0–35.2	29.0	3.0
Snout length	57.6	45.6–58.7	51.7	3.7

### Color in life

(Figs 1 and 3). Males. Head light brown above the eye, light blue to yellowish below the eye, with light blue markings on the cheek under the eye height. Black pupil, slightly pointed anteriorly. Iris ventral and anterior portion light blue. Black suborbital stripe absent. Light black vertical diffuse blotch on the posterior dorsal portion of head, anterior to dorsal-fin origin. Light blue markings at the anterior portion of the lateral line, sometimes surrounded by red diffuse blotches. Trunk and tail mainly iridescent blue. Five diffuse blotches in a longitudinal series in the middle portion of trunk and tail that may not be evident in colorful specimens, the second blotch is more conspicuous forming the “mid-lateral spot”. From these blotches, double diffuse vertical black lines extend to the ventral region. Scales on the dorsal portion of trunk and tail with black distal border and a basal black blotch, forming a black reticulate pattern with central portion of scales light iridescent blue. Ventral region whitish, although some males may present a yellowish hue. Dorsal fin with a grey dorsal distal band about the width of the pupil. Anterior portion of that fin, approximately first 8 dorsal-fin rays, hyaline to grey. Posterior portion with irregular white blotches, somehow elongated on the distal portion perpendicular to rays longitudinal axis. Basal portion of the posterior region of dorsal fin yellow and subdistal portion red. Caudal fin with rounded white blotches elongated in the distal portion. Basal central portion yellow, margins and distal portion red. Anal fin with white dots in the two basal thirds, distal one plain. Basal third area yellow, turning into orange in the medial third, and into red in the distal third. Pelvic fins with some light blue longitudinal stripes in the base parallel to longitudinal ray axis with a hyaline to light orange background. Pelvic fin hyaline to light orange.

Females. Head light brown, yellowish below the eye with light blue markings on the cheek. Black pupil, slightly pointed anteriorly. Iris black. Black suborbital stripe present. Light black vertical diffuse blotch on the posterior portion of head, anterior to dorsal-fin origin. Trunk and tail mainly brown yellowish. Five diffuse blotches in a longitudinal series in the middle portion of trunk and tail, the second blotch is more conspicuous forming the “mid-lateral spot”. From these blotches, double diffuse vertical black lines extend to the ventral region. Some scales in the middle portion of the trunk present light blue center forming longitudinal lines and surrounding the mid-lateral spot. Those lines of light blue dots are more conspicuous on the tail, where about six longitudinal lines of blue dots may be distinguished. The dorsal region presents a somehow marbled dark-brown pattern. Ventral region whitish. Dorsal, anal and caudal fin yellowish with orange distal portion, bearing hyaline irregular blotches, more irregularly shaped in the dorsal-fin, elongated in the caudal-fin and rounded in the anal-fin, where these are not present in the distal portion. Pelvic fins with some light blue longitudinal stripes in the base parallel to longitudinal ray axis with a hyaline to light orange background. Pelvic fin hyaline to light orange.

### Color in alcohol

(Fig 2). After fixation and conservation in alcohol the red and blue colorations are lost, remaining a similar pattern than live individuals but with a light brown color pattern and the black markings described before. Nine to 14 dark double vertical diffuse bars hardly discernible along mid-lateral surface of body, distributed between pectoral-fin base and vertical through last anal-fin ray origin, not visible in caudal peduncle. Number of vertical bars increase from small to large specimens. Mid-lateral spot present but without defined borders, on scales 8–10 or 9–11 of upper lateral line and of two scale rows below upper lateral line. Dark band in front of dorsal-fin origin, extending ventrally and slightly posteriorly. Head light brown, with diffuse dark markings ventral and posterior to eye and in upper portion of opercle. Isthmus and branchiostegal membrane light brown. Pectoral fin hyaline. Pelvic fin dark brown in males hyaline in females. Dorsal fin faint brown with small, narrow white blotches on posterior portion and grey in the anterior portion. Anal and caudal fins faint brown covered with rounded blotches. Suborbital band more conspicuous in females, not observable in males.

### Sexual dimorphism

Besides the differences in coloration described above and the presence of a hump, males are larger than females. No differences on meristic data were observed between the sexes.

### Distribution

**Fig 5:**
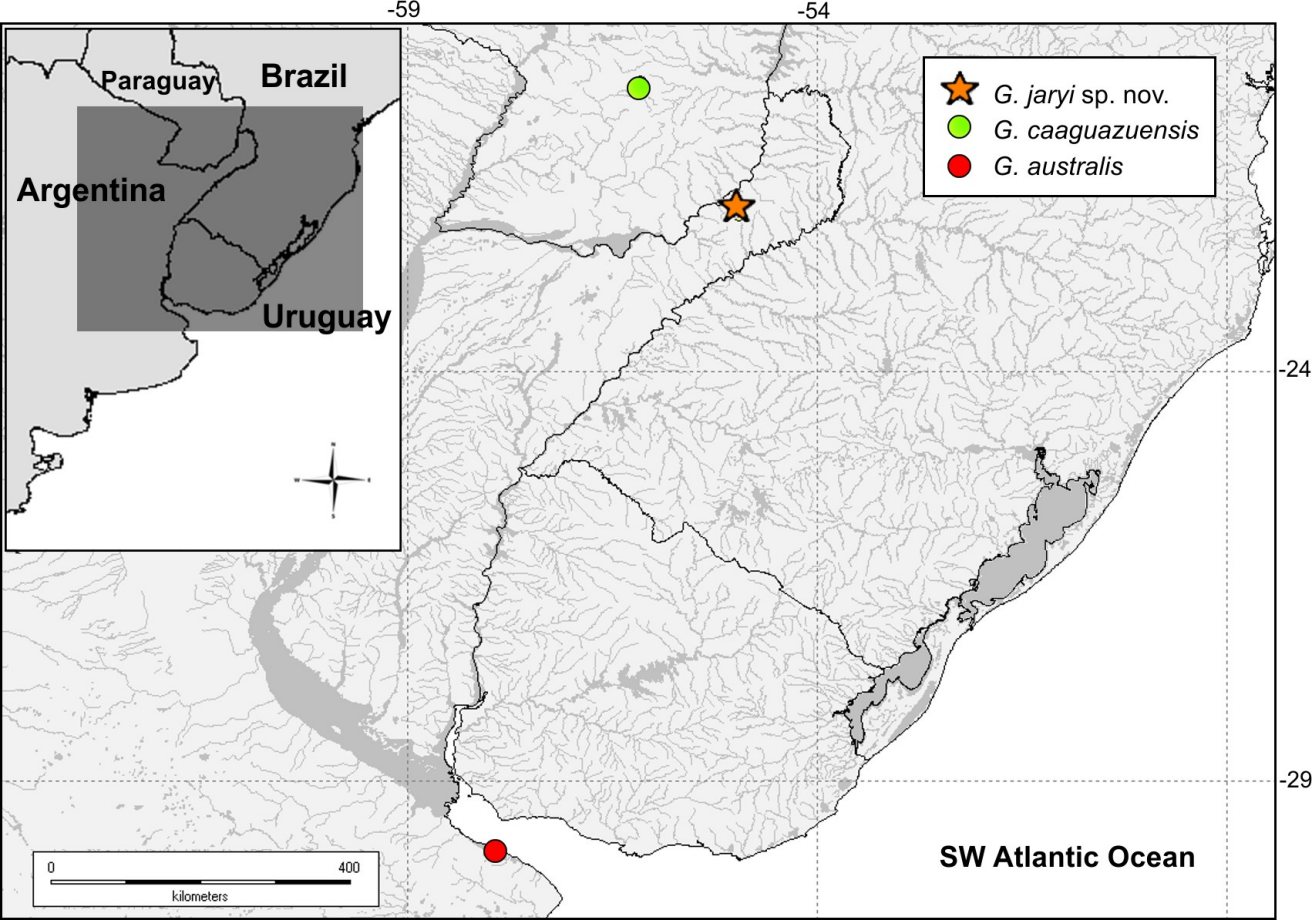
Known distribution of *Gymnogeophagus jaryi*, *G*. *caaguazuensis* and *G*. *australis*. Based only on material examined herein. Symbols may represent more than one locality. Image made with Diva-Gis.

*Gymnogeophagus jaryi* is known from several tributaries of the Southern Middle Paraná: the Cuña Pirú basin, the Garuhapé basin, and the Ñacanguazú basin in Argentina, Misiones and, based on photographs and mtDNA sequences it is also present in the Manduviyú, Pirapó and Ype Curú basins in Paraguay (Fig 5; [7]).

### Ecological notes

**Fig 6:**
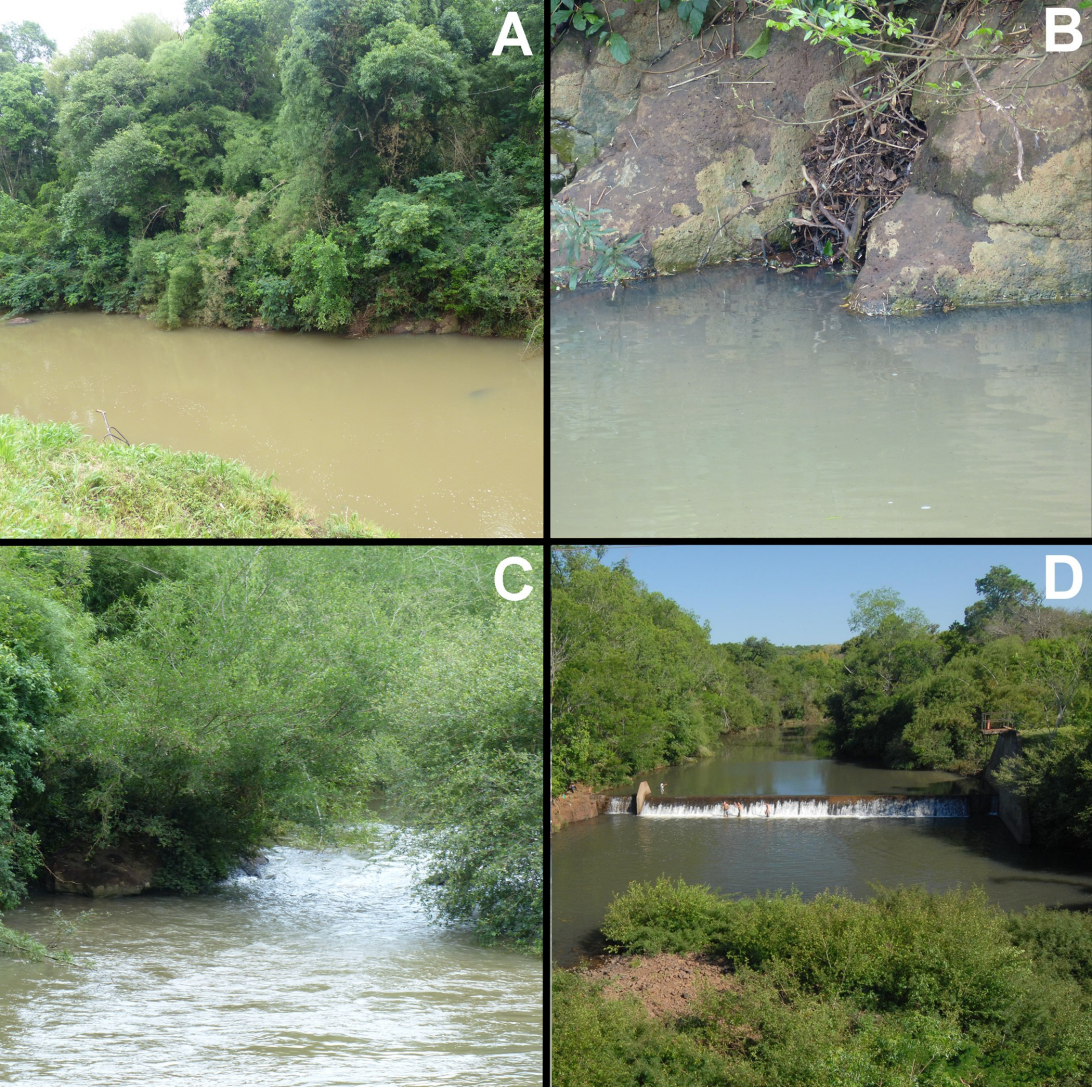
Habitats of *Gymnogeophagus jaryi*. at Middle Paraná River basin, Misiones, Argentina. (A, B, C) Type Locality at Cuñá Pirú stream;(B) rocks and driftwood in the margins; (C) rapids with marginal vegetation; D) Ñacanguazú stream. All photos show localities after rains in turbid conditions.

**Fig 7:**
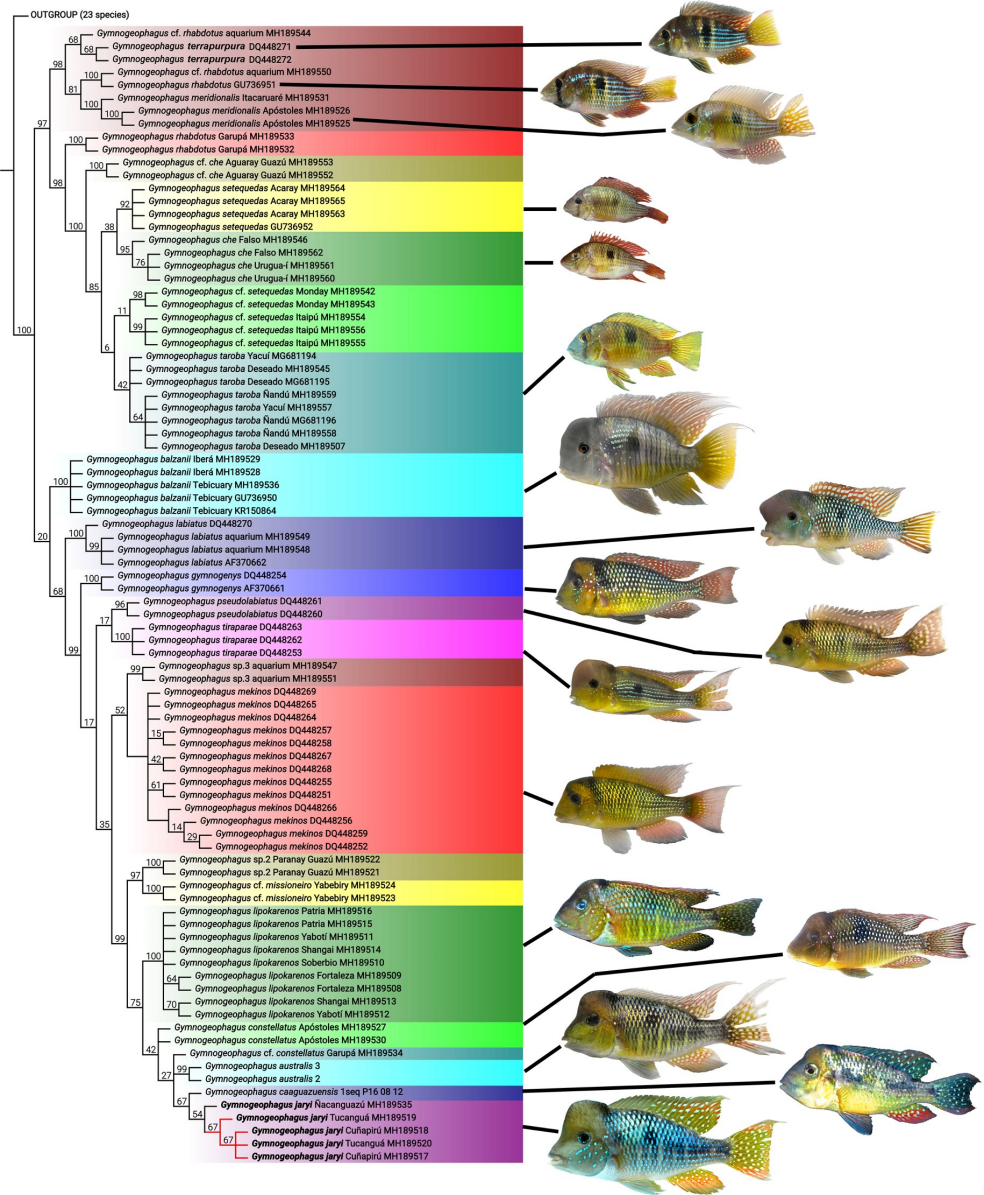
Phylogenetic relationships of *Gymnogeophagus jaryi* based on *cytb* marker. Analysis by parsimony under extended implied weighting. Numbers above branches denote GC values. Image of *G*. *caaguazuensis* and *G*. *constellatus* were taken and modified from their original description; [2, 11].

*Gymnogeophagus jaryi* inhabits streams with sandy and rocky bottom and abundant marginal vegetation (Figs 6 and 7). The climate on the mountain ridge in Aristóbulo del Valle is classified as warm and temperate. There is significant rainfall throughout the year in this area (average per year is 1,678 mm), and even the month with lowest precipitation, August, still has a considerable amount of rain (111 mm). The lowest precipitation months are July, August, and September, but the driest period and lowest water levels are in December, January, and February due to the marked seasonal change in temperature. The peaks in precipitation occur in May and October. The annual average temperature is 19.1°C, with 23.9ºC monthly average temperature in January and 14.3ºC in June [9]. Water level in the stream can have important changes (Fig 6). Water turbidity is variable among the year with peaks of turbidity after rains and increases in water visibility the rest of the year. Specimens are frequently found in the rocky areas or associated with marsh and marginal vegetation. The holotype was collected together with the female paratype shown in Fig 1 under the marginal vegetation and were with fry on November 2016. For further details on the sympatric species and habitat notes see Miquelarena *et al*. [8].

### Etymology

The specific epithet is derived from the Guaraní word “jarýi”, meaning grandmother. It is dedicated to the Non-Governmental Organization of “Abuelas de Plaza de Mayo”, created in 1977 whose objective is to locate and restore to their legitimate families all the children disappeared by the last Argentine dictatorship. A noun in apposition.

### Conservation status

*Gymnogeophagus jaryi* seems to be widely distributed in Southern tributaries of the middle Paraná basin and therefore the species can be categorized as Least Concern (LC) according to IUCN criteria [10].

### Molecular species delimitation

Both methods of molecular species delimitation (GMYC and bPTP) supported the independent species status of *G*. *jaryi*. The bPTP analysis (S3 Appendix) provided species delimitation results basically identical to the morphological determination while the GMYC analysis (not shown) did oversplit in several cases and supported two species-level clusters in several species (see [7]) including *G*. *jaryi*.

### Phylogenetic relationships

*Gymnogeophagus* was obtained as highly supported monophyletic unit (GC = 100), formed by two clades, the *G*. *rhabdotus* and the *G*. *gymnogenys* species groups. The *G*. *rhabdotus* group was obtained with high support (GC = 97), while the *G*. *gymnogenys* group was moderately well supported (GC = 20). Specimens of *G*. *jaryi* were obtained as a monophyletic unit (GC = 54), constituting the sister group of the single available specimen of *G*. *caaguazuensis* from the Paraguay River basin, and this clade is the sister group of *G*. *australis* from La Plata estuary basin, followed by *G*. cf. *constellatus* from the Middle Paraná basin in Misiones (Fig 7). Most of the rest of the species of the *G*. *gymnogenys* group inhabit the Uruguay River basin [7].

### Molecular divergence dating

Based on the BEAST molecular clock dating analysis *G*. *jaryi* diverged from *G*. *caaguazuensis* around 0.5 Ma and the divergence from *G*. *australis* and *G*. cf. *constellatus* dates to 0.7 Ma (S3 Appendix). *Gymnogeophagus jaryi* together with its sister species *G*. *caaguazuensis* are the youngest species in *Gymnogeophagus*. These two species have diverged within the last 500 Kya, while all other *Gymnogeophagus* species in the *G*. *gymnogenys* group are older than 1 Mya (most of them significantly older). Interestingly the only similarly young species in the whole genus are also found in the Middle Paraná basin (*G*. *setequedas* vs. *G*. *che* 0.8 Ma [7]).

## Discussion

*Gymnogeophagus jaryi* possesses the diagnostic characters of the genus *Gymnogeophagus* (*sensu* Reis, 1988). According to our phylogenetic analyses, *G*. *jaryi* is closely related to *G*. *caaguazuensis* and *G*. *australis*, species that share the light grey unspotted anterior portion of dorsal fin, although in some specimens it is slightly more yellowish grey in *G*. *australis* and slightly reddish grey in *G*. *jaryi*. A similar condition is observed among this genus in *G*. *taroba* (from the *G*. *rhabdotus* group), however in that case the anterior unspotted portion of dorsal fin is light grey orange to light grey red and it covers all the spiny portion of the fin and in *G*. *peliochelynion*, in which it is yellow [12]. The dorsal fin coloration within both species groups of *Gymnogeophagus* is one of the best character complexes that, in combination with other color pattern characters, enables virtually complete species determination [2, 3, 5]. The new species, *G*. *jaryi* described herein presents white blotches or short stripes on the posterior portion of the spiny portion and dots or spots (and thin lines distally) on the soft portion. The background coloration of the fin is yellow-orange ventrally and orange-red dorsally, except for the anterior part of the spiny portion, which is hyaline to grey. Several other *Gymnogeophagus* species have similarly colored and patterned dorsal fins, namely *G*. *lipokarenos*, *G*. *caaguazuensis*, *G*. *australis*, and *G*. *pseudolabiatus* among the *G*. *gymnogenys* group. The remaining species in the *G*. *gymnogenys* group have a markedly different coloration and spotting patterns of the dorsal fin: *G*. *tiraparae* shows a hyaline dorsal fin with two horizontal series of moderately elongated light blue dots between dorsal-fin spines, and a series of light blue stripes between soft rays, and a red ground color between the two series of dots; *G*. *constellatus* and *G*. *labiatus* have short wide stripes on spiny portion and long wide stripes on soft portion; *G*. *missioneiro* has long wide stripes on both portions; *G*. *mekinos* has the spiny portion without markings and the soft portion with only very few dots and distally immaculate; *G*. *gymnogenys* has much more numerous small or large round spots throughout the whole dorsal fin.

The new species seems to be widely distributed in the Southern portion of the Middle Paraná basin and thus, its conservation status is of less concern following the IUCN criteria. Nevertheless, the whole Middle Paraná and also its Southern portion includes notable endemism (i.e.: *Astyanax leonidas* Azpelicueta, Casciotta & Almirón, 2002, *A*. *troya* Azpelicueta, Casciotta & Almirón, 2002, *A*. *tupi* Azpelicueta, Mirande, Almirón & Casciotta, 2003, *Cnesterodon pirai* Aguilera, Mirande & Azpelicueta, 2009, *Cambeva ytororo* (Terán et al 2016)) [13, 14, 15, 16] which highlights the necessity for conservation policies in this region assuring the conservation of this ecosystem and its functions and the preservation of the ichthyological species in this region. For further details on the biogeography of this genus in the area see Rican *et al*. [7].

## Material and methods

Appropriate actions were taken to minimize pain or discomfort of fish, and this study was conducted in accordance with international standards on animal welfare, as well as being compliant with national regulations and the Comité Nacional de Ética en la Ciencia y la Tecnología of Argentina. Specimens were euthanized by immersion in an anesthetic solution (0.1% 2-phenoxyethanol), and then fixed in 4% formaldehyde for one week, washed in water for one day and transferred to a 70% ethanol solution for preservation. Prior to fixation DNA samples (fin clips) were taken and stored in absolute ethanol. These procedures are approved by the ethical use of animals of Instituto de Bio y Geociencias del NOA (IBIGEO) that consider animal welfare regulations. Collection permit was granted Ministerio de Ecología y Recursos Naturales Renovables de la Provincia de Misiones (Permits Nº 074/15, 509/07; 455/10; 474/13; 071/14; 003/16; 060/17) Descriptions of color patterns are based on photographs of live individuals. Counts and measurements were taken according to Reis & Malabarba [6] and Reis *et al*. [17]. Meristic data are presented listing all counts followed by the number of individuals in parentheses; counts of the holotype are marked with an asterisk. Vertebral counts are presented as abdominal + caudal, including the last half centrum. Measurements were taken with a caliper on the left side of the specimens. Measurements are expressed as percentages of the standard length (SL) except for subunits of the head which are recorded as percentages of the head length (HL). Scale row nomenclature follows Kullander [18]. Cleared and stained specimens (C&S) were prepared following Taylor & Van Dyke [19]. Type material is deposited in the ichthyological collections of Instituto de Bio y Geociencias del NOA (IBIGEO-I), Rosario de Lerma, Salta; Fundación Miguel Lillo (CI-FML), San Miguel de Tucumán; and Museo de La Plata collection (MLP), La Plata; all from Argentina. Additional examined material is provided as supplementary file.

### Species determination

We combine morphological species determination with post-hoc species delimitation using the molecular mtDNA cytochrome b (*cytb*) marker. Specimens were identified to species with the use of original descriptions, identification keys, and comparative material. Specimens that could not be identified to species level are reported as “*Gymnogeophagus* sp.” (possible new/unidentified species), specimens that were identified to species only with reservation are reported as “*Gymnogeophagus* cf. species” (a species that conforms to diagnosis with reservation and occurs outside its supposed distribution, possibly new) or “*Gymnogeophagus* aff. species” (closely related species, revealed by DNA phylogeny, possibly new). Our sampling for molecular analyses includes all described species of *Gymnogeophagus*, except *G*. *lacustris*, and several putatively new species. The dataset is that of [7] but includes two additional species, *G*. *australis* and *G*. *caaguazuensis*.

### Laboratory methods

Genomic DNA was extracted from ethanol-preserved gill or fin tissue using the JETQUICK Tissue DNA Spin Kit (Genomed, Germany) following standard protocol. The primers and reaction conditions of polymerase chain reaction (PCR) amplification are as in [7]. The products were analyzed in an ABI 3730XL automated sequencer (Applied Biosystems; Macrogen Inc., Korea). Contiguous sequences of the gene segments were created by assembling DNA strands (forward and reverse) using GENEIOUS v. 11.0.2 [20]. Nucleotide coding sequences were also translated into protein sequences to check for possible stop codons or other ambiguities. All newly generated sequences were submitted to GenBank under Accession numbersMK301452: *G*. *caaguazuensis*, MK301453: *G*. *australis*, MK301454: *G*. *australis*. Sequences were aligned using MUSCLE v. 3.8 [21], using the default settings.

### Phylogenetic analysis

A molecular phylogenetic analysis was performed, using the mitochondrial gene cytochrome b (*cytb*). Newly acquired sequences are from *G*. *caaguazuensis* and *G*. *australis*. The rest of the dataset is taken from Říčan et al. [7]. Alignments were trivial and the sequences did not imply gaps. Phylogenetic analyses were made by parsimony and Bayesian inference. Parsimony analyses were done with TNT under extended implied weighting [22, 23], assigning weights for each molecular character according to its own homoplasy (SEP, *sensu* Mirande [24]). Support was calculated through symmetric resampling, after 300 pseudoreplications of the dataset (with probability of change = 0.33), using sectorial searches and tree fusing on each pseudoreplicate [25]. Support values are given as differences between frequencies of the obtained groups and those of the most frequent alternative clades (GC-values) [26].

Bayesian inference analyses (BI) were performed in BEAST v.1.10.1 [27] with partitioning into codon positions (1st+2nd vs. 3rd). An optimal model of evolution according to Akaike criterion was selected using MrModeltest 2.2 [28] and PAUP* v. 4.0b10 [29]. The BI analysis using the Markov chain Monte Carlo (MCMC) simulation was run for 10 million generations with trees sampled and saved every 3,000 generations. Four independent analyses were performed to compare results of independent analyses. The analyses were run at the freely available Cipres server. The first 10% of trees from each run were discarded as burn-in. Convergence of the runs was estimated with the use of graphical visualization and diagnostics (especially the effective sample size; ESS) in Tracer v. 1.8.4 [30]. The remaining trees were used for reconstruction of the 50% majority-rule consensus tree with posterior probability (PP) values of the branches.

### Molecular clock dating analyses in BEAST

For divergence time estimation we used the Bayesian Evolutionary Analysis by Sampling Trees (BEAST) software package version v.1.10.1 [27] with parameters as above using the relaxed molecular clock model with lognormal distribution of rates and for tree prior the coalescent model with constant size. The calibration of the molecular clock was performed as an indirect secondary calibration based on the fossil *Gymnogeophagus eocenicus* Malabarba, Malabarba & Del Papa, 2010 [4] and the dated phylogeny of Musilová et al. [31]. Based on the results of the cited study the calibration date was set to 16 Ma with SD = 1 at the node uniting the two main groups within *Gymnogeophagus* (the *rhabdotus* and *gymnogenys* groups).

The analyses were also run at the freely available Cipres server. Runs were checked for convergence with Tracer v.1.10.1 [30]. Four well converged runs were combined in LogCombiner v.1.10.1 with a burn-in of 10% for each of the data partition schemes. The final tree for each data partition scheme was produced from these data with TreeAnnotator v.1.8.4.

### Species delimitation analyses using GMYC and PTP

We employed the General Mixed Yule Coalescent (GMYC) and Poisson tree processes (PTP) analyses for molecular species delimitation using the *cytb* marker. Both methods were designed for delimiting species based primarily on single molecular markers (hence where multilocus coalescent-based methods are not applicable). The General Mixed Yule Coalescent (GMYC) model [32, 33] is frequently used in empirical studies [34, 35, 36, 37, 38] and the newer Poisson tree processes (PTP) model [39] has been shown to even outperform the GYMC method where distances between species are small. Both methods outperform OTU-picking methods (relying on simple sequence similarity thresholds) and are more robust to cases where the barcoding gap is absent [39].

PTP and GMYC analyses were run at the freely available web interface (http://species.h-its.org/). The bPTP analysis was run both on the MrBayes tree and on the ultrametric BEAST tree in order to compare results (see [39]) and in the latter case to make the bPTP result directly comparable to the GMYC result.

### Nomenclatural acts

The electronic edition of this work follows the requirements of the International Code of Zoological Nomenclature, and hence the new names contained herein are available under that Code from the electronic edition of this article. This published work and the nomenclatural acts it contains have been registered in ZooBank, the online registration system for the ICZN. The ZooBank LSIDs (Life Science Identifiers) can be resolved and the associated information viewed through any standard web browser by appending the LSID to the prefix “http://zoobank.org/”.”. The LSID for this publication is: urn:lsid:zoobank.org:pub:4E724041-85FE-44CA-9A4F-EB42E62BB672.

## Supporting information

S1 Appendix List of material examined. (PDF)

S2 Appendix Character matrix used for the phylogenetic analysis. (TNT)

S3 Appendix Phylogenetic relationships and molecular species delimitation from BEAST analysis. (PDF)

## References

[1] Fricke R, Eschmeyer WN, Fong JD. 2018. [cited 18 Sep 2018]. SPECIES BY FAMILY/SUBFAMILY. Available from: http://researcharchive.calacademy.org/research/ichthyology/catalog/SpeciesByFamily.asp

[2] Malabarba LR, Malabarba MC, Reis RE. Descriptions of five new species of the Neotropical cichlid genus Gymnogeophagus Miranda Ribeiro, 1918 (Teleostei: Cichliformes) from the río Uruguay drainage. Neotrop. Ichthyol. 2015;13:637–662. 10.1590/1982-0224-20140188

[3] Casciotta J, Almirón A, Piálek L, Říčan O. Gymnogeophagus taroba (Teleostei: Cichlidae), a new species from the río Iguazú basin, Misiones, Argentina. Historia Natural (Tercera Serie). 2017;7:5–22.

[4] Malabarba MC, Malabarba LR, Del Papa C. Gymnogeophagus eocenicus, n. sp. (Perciformes: Cichlidae), an eocene cichlid from the Lumbrera Formation in Argentina. J Vertebr Paleontol. 2010;30:341–350. 10.1080/02724631003618348

[5] Loureiro M, Zarucki M, Malabarba LR, & González-Bergonzoni I. A new species of Gymnogeophagus Miranda Ribeiro from Uruguay (Teleostei: Cichliformes). Neotrop Ichthyol. 2016;14(1):155–164. 10.1590/1982-0224-20150082

[6] Reis RE, Malabarba LR. Revision of the Neotropical cichlid genus Gymnogeophagus Ribeiro, 1918, with descriptions of two new species (Pisces, Perciformes). Rev Bras Zool. 1988;4:259–305. 10.1590/S0101-81751987000400002

[7] Říčan O, Říčanová Š, Dragová K, Piálek L, Almirón A, Casciotta J. Species diversity in Gymnogeophagus (Teleostei: Cichlidae) and comparative biogeography of cichlids in the Middle Paraná basin, an emerging hotspot of fish endemism. Hydrobiologia. 2018;1–24. 10.1007/s10750-018-3691-z

[8] Miquelarena AM, Protogino LC, Filiberto R, López HL. A new species of Bryconamericus (Characiformes: Characidae) from the Cuña-Pirú creek in north-eastern Argentina, with comments on accompanying fish. Aqua. 2002;6(2):69–82.

[9] Climate-Data.org. 2018. [cited 18 Sep 2018]. Clima: Aristóbulo del Valle. In: Climate data for cities worldwide. Available from: https://es.climate-data.org/location/145265/

[10] IUCN Red List. Categories & Criteria: Version 3.1. IUCN Species Survival Commission, Gland, Switzerland. 2001. Accessed at http://www.iucnredlist.org/static/categories_criteria_3_1#categories

[11] Staeck W. Gymnogeophagus caaguazuensis sp. n.—a new species of cichlid fish (Teleostei: Perciformes: Cichlidae) from the drainage of the lower río Paraguay in Paraguay. Zoologische Abhandlungen (Dresden). 2006; 56:99–105.

[12] Turcati A, Serra-Alanis WS, Malabarba LR. A new mouth brooder species of Gymnogeophagus with hypertrophied lips (Cichliformes: Cichlidae). Neotrop. ichthyol. [online]. 2018,16(4): e180118 [cited 2018-12-14]. Available from: <http://www.scielo.br/scielo.php?script=sci_arttext&pid=S1679-62252018000400218&lng=en&nrm=iso>. Epub Dec 06, 2018. ISSN 1679-6225. 10.1590/1982-0224-20180118.

[13] Azpelicueta MM, Casciotta JR, Almirón AE. Two new species of the genus Astyanax (Characiformes, Characidae) from the Paraná river basin in Argentina. Revue Suisse de Zoologie. 2002;109(2):243–259.

[14] Azpelicueta MM, Mirande JM, Almirón AE, Casciotta JR. A new species of Astyanax (Characiformes, Characidae) from Paraná river basin in Argentina. Revista del Museo de La Plata. 2003;15(166):1–12.

[15] Aguilera G, Mirande JM, Azpelicueta MM. A new species of Cnesterodon (Cyprinodontiformes: Poeciliidae) from a small tributary of arroya Cuñá-Pirú, río Paraná basin, Misiones, Argentina. Zootaxa. 2009;2195:34–42.

[16] Terán GE, Ferrer J, Benitez M, Alonso F, Aguilera G, Mirande JM. Living in the waterfalls: A new species of Trichomycterus (Siluriformes: Trichomycteridae) from Tabay stream, Misiones, Argentina. PloS One. 2017;12(6):e0179594 10.1371/journal.pone.0179594 28640842 PMC5480901

[17] Reis RE, Malabarba LR, Pavanelli CS. Gymnogeophagus setequedas, a new cichlid species (Teleostei: Labroidei) from middle rio Paraná system, Brazil and Paraguay. Ichthyol Explor Freshw. 1992;3:265–272.

[18] Kullander SO. Heroina isonycterina, a new genus and species of cichlid fish from Western Amazonia, with comments on cichlasomine systematics. Ichthyol Explor Freshw. 1996;7:149–172.

[19] Taylor WR, Van Dyke GC. Revised procedures for staining and clearing small fishes and other vertebrates for bone and cartilage study. Cybium. 1985;9:107–119.

[20] Kearse M, Moir R, Wilson A, Stones-Havas S, Cheung M, Sturrock Shane, et al Geneious Basic: an integrated and extendable desktop software platform for the organization and analysis of sequence data. Bioinformatics. 2012;28(12):1647–1649. 10.1093/bioinformatics/bts199 22543367 PMC3371832

[21] Edgar RC. MUSCLE: multiple sequence alignment with high accuracy and high throughput. Nucleic acids research. 2004;32:1792–1797. 10.1093/nar/gkh340 15034147 PMC390337

[22] Goloboff PA. Estimating character weights during tree search. Cladistics. 1993;9:83–91. 10.1006/clad.1993.1003 34929936

[23] Goloboff PA. Extended implied weighting. Cladistics. 2014;30:260–72. 10.1111/cla.12047 34788977

[24] Mirande JM. Morphology, molecules and the phylogeny of Characidae (Teleostei, Characiformes). Cladistics. 2018;1–19. 10.1111/cla.12345 34622981

[25] Goloboff PA. Analyzing large data sets in reasonable times: solutions for composite optima. Cladistics. 1999;15:415–428. 10.1111/j.1096-0031.1999.tb00278.x 34902941

[26] Goloboff PA, Farris JS, Källersjö M, Oxelman B, Ramírez MJ, Szumik CA. Improvements to resampling measures of group support. Cladistics. 2003;19:324–332. 10.1111/j.1096-0031.2003.tb00376.x

[27] Drummond AJ, Rambaut A. BEAST: Bayesian evolutionary analysis by sampling trees. BMC Evol Biol. 2007;7:1–8. 10.1186/1471-2148-7-1 17996036 PMC2247476

[28] Nylander JAA. 2004 MrModeltest Version 2. Program Distributed by the Author. Evolutionary Biology Centre, Uppsala University, Uppsala.

[29] Swofford DL. 2002 PAUP*: Phylogenetic analysis using parsimony (*and other methods). v.4.0b10. Sinauer & Associates, Sunderland, Massachusetts.

[30] Rambaut A, Drummond AJ. 2007. Tracer 1.5.0. Available on internet at: http://beast.bio.ed.ac.uk/Tracer

[31] Musilová Z, Říčan O, Říčanová S, Janšta P, Gahura O, Novák J. Phylogeny and historical biogeography of trans-Andean cichlid fishes (Teleostei: Cichlidae). Vertebr Zool. 2015;65(3):333–350.

[32] Pons J, Barraclough TG, Gomez-Zurita J, Cardoso A, Duran DP, Hazell S, et al Sequence-based species delimitation for the DNA taxonomy of Undescribed insects. Syst Biol. 2006;55:595–609. 10.1080/10635150600852011 16967577

[33] Fujisawa T, Barraclough TG. Delimiting Species Using Single-Locus Data and the Generalized Mixed Yule Coalescent Approach: A Revised Method and Evaluation on Simulated Data Sets. Syst Biol. 2013;62(5):707–724. 10.1093/sysbio/syt033 23681854 PMC3739884

[34] Fontaneto D, Herniou EA, Boschetti C, Caprioli M, Melone G, Ricci C, et al Independently evolving species in asexual bdelloid rotifers. PLoS Biol. 2007;5:e87 10.1371/journal.pbio.0050087 17373857 PMC1828144

[35] Monaghan MT, Wild R, Elliot M, Fujisawa T, Balke M, Inward DJG, et al Accelerated species inventory on madagascar using coalescent-based models of species delineation. Syst Biol. 2009;58:298–311. 10.1093/sysbio/syp027 20525585

[36] Carstens BC, Dewey TA. Species delimitation using a combined coalescent and information-theoretic approach: an example from North American Myotis bats. Syst Biol. 2010;59:400–414. 10.1093/sysbio/syq024 20547777 PMC2885268

[37] Vuataz L, Sartori M, Wagner A, Monaghan MT. Toward a DNA Taxonomy of Alpine Rhithrogena (Ephemeroptera: Heptageniidae). Using a Mixed Yule-Coalescent Analysis of Mitochondrial and Nuclear DNA. PLoS One. 2001;6:e19728 10.1371/journal.pone.0019728 21611178 PMC3096624

[38] Powell JR. Accounting for uncertainty in species delineation during the analysis of environmental DNA sequence data. Methods Ecol Evol. 2012;3:1–11. 10.1111/j.2041-210X.2011.00122.x

[39] Zhang J, Kapli P, Pavlidis P, Stamatakis A. A general species delimitation method with applications to phylogenetic placements. Bioinformatics. 2013;29:2869–2876. 10.1093/bioinformatics/btt499 23990417 PMC3810850

