# Supplementary material for: Description of a new species of the Neotropical cichlid genus Gymnogeophagus Miranda Ribeiro, 1918 (Teleostei: Cichliformes) from the Middle Paraná basin, Misiones, Argentina
Source: PLoS One. 2019 Feb 13;14(2):e0210166. doi: 10.1371/journal.pone.0210166 (PMC6374015; doi:10.1371/journal.pone.0210166)
Supplement: S1 Appendix — (PDF) [file pone.0210166.s001.pdf]

EXAMINED MATERIAL

Institutional abbreviations:

CI-FML, ichthyological collection Fundación Miguel Lillo, Tucumán, Argentina

IBIGEO-I, ichthyological collection Instituto de Bio y Geo Ciencias del NOA, Salta, Argentina.

MLP, Museo de La Plata, La Plata, Argentina

MHNM, Museo Nacional de Historia Natural de Montevideo, Uruguay

ZVC-P Facultad de Ciencias, Universidad de la República, Montevideo.

Other abbreviations: C&S, cleared and stained;

Gymnogeophagus australis. All from Argentina, Buenos Aires: IBIGEO-I 466, 4, 3.5-8.5 mm, Isla Santiago, Río de La Plata (approx. 34°50'05.1"S 57°55'33.1"W), Walldbillig, Acuña y Bozza, II-2018. IBIGEO-I 467, 1, 7.0 mm SL, La Balandra, Río de la Plata (approx. 34°55'44.5"S 57°43'04.4"W), García, Bozza, III-2018. MLP 11380, 1, 117.0 mm, Río de La Plata frente a la Isla Paulino (34º50'01.8 S 57º52'49.5 W), Nicolás Tizzio, I-2017.

Gymnogeophagus caaguazuensis. IBIGEO-I 464, 6 ex, 45.3-58.3 mm, upper río Tebicuary-mi (approx. 25°28'S, 56°10'W), tributary to río Tebicuary (drainage of the lower río Paraguay), at ruta No. 7 approx.25 km east of Cnel. Oviedo in the province of Caaguazú in Paraguay.

Gymnogeophagus constellatus. All from Argentina, Misiones province, río Uruguay basin: MLP 11285, 8 ex., 31,4-115,4 mm SL, arroyo Itacaruaré (27º52'33.8"S - 55º16'35.1"W). April 2000. MLP 11286, 5 ex., 34,9-83,1 mm SL, arroyo Itacaruaré (27º52'33.8"S - 55º16'35.1"W). October 2009. MLP 11287, 1 ex., 67,5 mm SL, arroyo Chimiray-Miní, (27º50'50.2"S - 55º45'53.9"W). December 2007

Gymnogoephagus jaryi. MLP 11365, 29 ex., 46.2-116.6 mm SL, río Paraná basin, Misiones, arroyo Ñacanguazú (20º07'14.1"S--55º22'22.1" W). Col: Rican et al., 23/10/2009.

Gymnogeophagus mekinos: MHNM 3511, 2 ex. 97.2-105.1 mm SL, Paso de la Arena, Río Santa Lucía Chico, 34°01'51"S 56°09'45"W, Florida, URUGUAY, coll. W.S. Serra & M. Pérez, 11/I/2014; MHNM 4009, 4 ex. 49.9-121.3 mm SL, Arroyo Rocha, Parque La Estiva, Ciudad de Rocha, 34°29'38"S 54°20'43"W, Rocha, URUGUAY, coll. W.S. Serra, 22/I/2018.

MHNM 4069, 1 ex. 54.6 mm SL, laguna marginal del Aº Rocha, 200 m al N de Ruta 9, 34°30'58"S 54°20'57"W, Rocha, URUGUAY, coll. W.S. Serra, 11/III/2018; MHNM 4075, 3 ex. 55.6-103.4 mm SL, Arroyo del Paraná, 33°33'45"S 55°41'35"W, Florida, URUGUAY, coll. W.S. Serra, N. Ríos & C. Clavijo, 12/I/2018; ZVC-P 3699, 2 ex. 47.3-82.1 mm SL, Arroyo Laureles, 31°15'17"S 56°03'14"W, Tacuarembó, URUGUAY, II/1954; ZVC-P 3713, 2 ex. 52.7-82.0 mm SL, Arroyo Laureles, 31°15'17"S 56°03'14"W, Tacuarembó, URUGUAY, II/1954; ZVC-P 4058, 1 ex. 91.7 mm SL, Arroyo Las Higueras, 32°56'44"S 56°02'20"W, Durazno, URUGUAY, coll. E. Messner, 5/X/1959; ZVC-P 6696, 1 ex. 76.9 mm SL, Paraje 329, Río Negro, 32°26'41"S 55°26'00"W, Durazno, URUGUAY, coll. I. González-Bergonzoni, 25/VIII/2005; ZVC-P 9663, 3 ex. 50.4-73.5 mm SL, Laguna del Medio, Ciudad de Florida, 34°05'50"S 56°12'09"W, Florida, URUGUAY, coll. W.S. Serra, 30/VIII/2009; ZVC-P 11072, 2 ex. 56.4-61.2 mm SL, Embalse de Rincón del Bonete, Río Negro, 32°49'S 56°25'W, Tacuarembó, URUGUAY, coll. I. González-Bergonzoni, 1/XI/2008; ZVC-P 11706, 3 ex. 41.5-108.4 mm SL, Paso Arriera, 31°58'48"S 54°33'17"W, Rivera, URUGUAY, coll. M. Loureiro, A. Duarte, W.S. Serra & S. Starecek, 2/VIII/2013.

***Gymnogeophagus labiatus*:** MHNM 4074, 5 ex. 56.8-126.9 mm SL, Arroyo Gutiérrez y Ruta 8, 33°31'22"S 54°34'10"W, Lavalleja, URUGUAY, coll. W.S. Serra, N. Ríos & C. Clavijo, 11/I/2018; MHNM 4076, 3 ex. 44.0-96.6 mm SL, Río Cebollatí, 33°55'23"S 55°10'34"W, Lavalleja, URUGUAY, coll. W.S. Serra, N. Ríos & C. Clavijo, 11/I/2018; ZVC-P 6281, 2 ex. 42.2-62.7 mm SL, Arroyo de los Chanchos, embalse de Villa Serrana, 34°19'16"S 54°59'18"W, Lavalleja, URUGUAY, coll. F. Scasso, M. Loureiro, F. Quintans & J. Sawchick, 3/II/2001; ZVC-P 6423, 2 ex. 39.2-78.7 mm SL, Arroyo de las Averias, Paso del Aguila, 33°13'25"S 55°00'01"W, Treinta y Tres, URUGUAY, coll. F. Scasso, M. Loureiro, F. Quintans & J. Sawchick, 4/II/2001; ZVC-P 6840, 12 ex. 21.6-55.6 mm SL, Arroyo Yerbalito, Sierra de Ríos, 32°11'35"S 53°45'02"W, Cerro Largo, URUGUAY, coll. F. Scasso, M. Loureiro, F. Quintans & J. Sawchik, 7/II/2001; ZVC-P 7053, 5 ex. 51.1-72.1 mm SL, cantera de piedra junto a la Ruta 7, 33°22'16"S 55°09'10"W, Treinta y Tres, URUGUAY, coll. F. Scasso, M. Loureiro, F. Quintans & J. Sawchick, 3/II/2001; ZVC-P 7057, 2 ex. 48.9-72.1 mm SL, Río Olimar, 33°15'22"S 54°23'06"W, Treinta y Tres, URUGUAY, coll. F. Scasso, M. Loureiro, F. Teixeira & N. Marchand, 21/II/2001; ZVC-P 11948, 1 ex. 93.7 mm SL, Paso San Diego, Río Yaguarón, 31°57'57"S 53°54'52"W, Cerro Largo, URUGUAY, coll. M. Loureiro, F. Teixeira, S. Clavijo & M. Zarucki, IX/2007.

Gymnogeophagus lipokarenos: all lots from Argentina, Misiones province, Uruguay river basin: MLP 11251, 2 exs., 82,7-86,2 mm SL, arroyo Paraiso (27°2'52,49"S 54°5'54,97"W) 22.11.2016 (fig. 1-2). MLP 11252, 3 exs., 116,3-127,0 mm SL, lower arroyo Paraiso (27°9'26,58"S 54°4'0,46"W) 24.11.2016. MLP 11253, 4 exs., 72,8-127,4 mm SL, arroyo Guerrero (arroyo López) (27°45'57,4"S 55°09'33,7"'W), 03.12.2007 (fig. 3). MLP 11254, 2 exs., 80,0-87,3 mm SL, arroyo Fortaleza (26°45'56,6"S 54°10'57,4"W),

01.12.2007. MLP 11255, 5 exs., 61,8-87,2 mm SL, arroyo Shangai (27°28'13,8"S 54°41'24,5"W). MACN-Ict 12306, 6 exs., 61,6-94,5 mm SL, arroyo El Saltito, Salto Golondrinas (27°7'50,34"S 54°29'22,54"W) 23.11.2016 (fig. 4). MACN-Ict 12304, 2 exs., 103,8-117,0 mm SL, arroyo El Saltito, Salto Caracol (27°9'27,40"S 54°38'19,53"W), 25.11.2016. MACN-Ict 12305, 1 ex., 133.7 mm SL, arroyo Toro (26°36'32,8"S 53°44'13,9"W), 10.12.2014. IBIGEO-I 448, 5 exs., 88.0-118.6 mm SL, arroyo Toro (26°36'32,8" S, 53°44'13,9" W), 01.12.2016. IBIGEO-I 450, arroyo Melo (27°25'2,67"S 54°42'7,93"W), 13.11.2016 (fig. 7). CI-FML 7264, 4 ex., 39,2-108,8 SL, same collecting data as previous lot.

Gymnogeophagus gymnogenys: MHNM 4073, 3 ex. 55.0-97.1 mm SL, Arroyo Gutiérrez y Ruta 8, 33°31'22"S 54°34'10"W, Lavalleja, URUGUAY, coll. W.S. Serra, N. Ríos & C. Clavijo, 11/I/2018; MHNM 4077, 1 ex. 71.0 mm SL, Río Cebollatí, 33°55'23"S 55°10'34"W, Lavalleja, URUGUAY, coll. W.S. Serra, N. Ríos & C. Clavijo, 11/I/2018; ZVC-P 5861, 5 ex. 50.8-102.4 mm SL, Arroyo India Muerta, 34°11'59"S 54°15'39"W, Rocha, URUGUAY, XII/2004; ZVC-P 7044, 5 ex. 20.3-85.6 mm SL, Cañada Tres Boliches y Ruta 8, 32°16'31"S 54°09'50"W, Cerro Largo, URUGUAY, II/2001; ZVC-P 8952, 1 ex. 91.1 mm SL, embalse de India Muerta, 33°55'32"S 54°14'57"W, Rocha, URUGUAY, coll. F. Scasso, A. D'Anatro & N. Marchand, 8/III/2001; ZVC-P 8959, 2 ex. 43.5-78.9 mm SL, Río Yaguarón, 32°35'20"S 53°20'12"W, Cerro Largo, URUGUAY, coll. F. Scasso, A. D'Anatro & N. Marchand, 10/III/2001; ZVC-P 9330, 2 ex. 78.0-95.2 mm SL, Arroyo Yerbal Chico, Quebrada de los Cuervos, Treinta y Tres, URUGUAY, coll. M. Loureiro & M. Zarucki, 16/XII/2008; ZVC-P 12893, 4 ex. 63.5-91.7 mm SL, Arroyo Molles próximo a Ruta 8, 33°34'20"S 54°30'02"W, Lavalleja, URUGUAY, coll. G. Eguren, J. Cabrera & G. Silva, XI/2010;

Gymnogeophagus tiraparae: ZVC-P 3703, paratipos, 3 ex. 59.9-80.4 mm SL, Río Negro y Ruta 6, Paraje 329, 32°26'44"S 55°26'03"W, Durazno, URUGUAY, coll. F. Achaval, X/1998; ZVC-P 7870, holotipo, 1 ex. 98.8 mm SL, Río Tacuarembó, Pueblo Ansina, 31°53'01"S 55°28'38"W, Tacuarembó, URUGUAY, coll. S. Oviedo, I. Gonzalez, F. Teixeira, A. Danatro & M. Loureiro, XI/2005; ZVC-P 11624, 5 ex. 56.8-78.5 mm SL, Paso San Borja, Río Yí, 33°23'50"S 56°24'10"W, Durazno, URUGUAY, 5/II/2007; ZVC-P 11987, 10 ex. 51.9-76.6 mm SL, Río Negro, correderas del 329, 32°27'53"S 55°25'18"W, Durazno, URUGUAY, coll. M. Loureiro, A. Duarte, W.S. Serra & J. Bessonart, 15/I/2013.

Gymnogeophagus pseudolabiatus: MHNM 4010, 7 ex. 55.3-95.3 mm SL, Arroyo Pintado y Ruta 30, 30°26'16"S 56°26'59"W, Artigas, URUGUAY, coll. W.S. Serra & N. Ríos, 2/XII/2017; MHNM 4078, 4 ex. 55.3-95.3 mm SL, Paso Ferrayen, 30°26'23"S 56°25'22"W, Artigas, URUGUAY, coll. W.S. Serra & N. Ríos, 2/XII/2017; MHNM 1615, 7 ex. 55.1-77.3 mm SL, A° Pintado Grande, Est. Stolowac, aprox. 30°46'S 56°14'W, Artigas, URUGUAY,

coll. R. Carrera, 13 a 29/III/1970; MHNM 1616, 13 ex. 29.7-73.4 mm SL, A° Pintado Grande, Est. Stolowac, aprox. 30°46'S 56°14'W, Artigas, URUGUAY, coll. R. Carrera, 13 a 29/III/1970; ZVC-P 7455, 2 ex. 83.8-93.6 mm SL, Arroyo Catalán Grande y Ruta 30, 30°50'27"S 56°14'20"W, Artigas, URUGUAY, coll. M. Loureiro, I. González, F. Teixeira & F. Quintans, II/2006; ZVC-P 10011, 11 ex. 25.0-86.4 mm SL, afluente de la Cañada Honda, 30°27'46"S 56°51'24"W, coll. M. Loureiro, I. González, F. Teixeira & F. Quintans, VIII/2006; ZVC-P 12616, 8 ex. 36.2-91.5 mm SL, Laguna Salamanca, Río Cuareim, Paraje Sepulturas, 30°46'36"S 56°02'16"W, Artigas, URUGUAY, coll. A. Duarte, W.S. Serra, M. Loureiro & L. Ziegler, 1-4/XII/2012; ZVC-P 12684, 2 ex. 94.9-97.2 mm SL, Arroyo Cuaró Grande y Ruta 4, 30°46'57"S 56°47'05"W, Artigas, URUGUAY, coll. M. Loureiro, J. Bessonart, W.S. Serra & L. Montes de Oca, 11/VIII/2013.

***Gymnogeophagus peliochelynion*.**: MHNM 3711, 4 ex. 50.0-79.9 mm SL, Río Arapey Chico y Ruta 4, 31°02'07"S 56°53'50.21"W, Salto, URUGUAY, coll. F. Teixeira, A. D'Anatro, I. González, S. Oviedo & M. Loureiro, 22/XI/2005; ZVC-P 7016, 16 ex. 43.3-94.1 mm SL, Río Arapey Chico y Ruta 4, 31°02'07"S 56°53'50.21"W, Salto, URUGUAY, coll. F. Teixeira, A. D'Anatro, I. González, S. Oviedo & M. Loureiro, 22/XI/2005; ZVC-P 13210, 1 ex. 76.3 mm SL, Río Arapey, Colonia Lavalleja, Paso Elías, 31°02'50"S 57°00'46"W, Salto, URUGUAY, coll. M. Loureiro, A. Duarte, S. Serra, J. Bessonart & S. Paullier, 17/XII/2014; ZVC-P 13057, 2 ex. 65.3-90.2 mm SL, Arroyo Sopas, Paso del Cementerio, 31°23'34"S 56°42'31"W, Salto, URUGUAY, coll. M. Loureiro, A. Duarte & S. Serra, 18/XII/2014; ZVC-P 13084, 4 ex. 46.4-79.7 mm SL, Cañada de la Tapera, 31°09'02"S 56°13'25"W, Salto, URUGUAY, coll. M. Loureiro, A. Duarte, S. Serra, J. Bessonart & S. Paullier, 13/XII/2014; ZVC-P 13118, 6 ex. 56.3-77.2 mm SL, Río Arapey, Picada Sarandí, 31°03'52"S 56°22'44"W, Salto, URUGUAY, coll. M. Loureiro, A. Duarte, S. Serra, J. Bessonart & S. Paullier, 15/XII/2014; ZVC-P 13139, 2 ex. 52.7-57.7 mm SL, Arroyo Mataojo Grande, Paso de la Herrería, 31°11'45"S 56°36'11"W, Salto, URUGUAY, coll. M. Loureiro, A. Duarte, S. Serra, J. Bessonart & S. Paullier, 14/XII/2014.

***Gymnogeophagus* sp.** MLP 3954, 1 ex. 87 mm, 32°59'51.61"S 58°30'5.09"W Gualeguaychú, Entre Ríos, Argentina, coll. Daneri. MLP-6693, 2 ex. 63-67 mm, Lago parque Belgrano, Santa Fe, Argentina, coll. M. Galván & G. Martín, 18/7/1960. MLP 1014, 1 ex. 92mm, Ensenada, Buenos Aires, Argentina. MLP 7268, 2 ex, Madrejón Flores Santa Fe, Argentina, coll A. Bonetto, 11/9/1961. MLP 7895, 1 ex, Santa Fe, Argentina, coll A. Bonetto, 2/3/1967. MLP 3955, 1 ex, 45 mm, Gualeguaychu, Entre Ríos, Argentina, coll I. Daneri, 6/10/1945. MLP 4714, 1 ex, 47 mm, Entre Ríos, Argentina, coll I Daneri, 6/6/1947. MLP 9332, 1 ex, 91 mm, Laguna del arbolito Laborde, Cordoba, coll Haro & Bistoni". MLP 9649, 107 ex 29 -143 mm, Arroyo grande, Concordia, Entre Ríos, Argentina. coll E. Mac Donagh 3/5/1943. MLP 8395, 1 ex 98mm, Lago frente Club Tifón, Santa Fe, Argentina. Coll P. Ceresole 17/3/1980. MLP 6082, 2 ex, Laguna Estevez, Entre

Ríos, Argentina. Coll M Galvan & E. Martín 4/8/1952. MLP 8373, 1ex, Bañados Río Colastiné Sur, Santa Fe, Argentina. 1956. MLP 6824, 1ex, Rosario, Santa Fe, Argentina. MLP 10388, 45 ex, arrollo mendizco (boca) Gualeguychú, Entre Ríos, Argentina. Coll Baigún, Brancolini, Oldani, Sire, 23/5/2008.

Gymnogeophagus terrapurpura: all lots from Argentina, Entre Ríos province, Colón county, Uruguay river basin: MACN-ict 12248, 75-77 mm SL. Arroyo de la Leche at RN 135, near the city of Colón, 32°14'42"S 58°8'58"W, coll. H.G. Fernández, 28.01.2016. MLP 11227, 62 mm SL. Municipio Villa Elisa, balneario municipal, Arroyo Perucho Verna, 32°10'27"S 58°18'33"W. coll. A. Puentes, 09.2016. ZFMK 39780, 7.5 mm SL. Municipio Ubajay, estancia Los Monigotes, Arroyo San Benito, 31°49’64”S 58°10’74”W. coll. S. Koerber, R. Filiberto, J.O. Fernández Santos, 02.02.2002.

* Additional examined material listed in Casciotta *et al*. (2017):

Casciotta, J., A. Almirón, L. Piálek & O. Říčan, 2017. *Gymnogeophagus taroba* (Teleostei: Cichlidae), a new species from the rı´o Iguazu´ basin, Misiones, Argentina. Historia Natural (Tercera Serie) 7: 5–22.
