# Supplementary material for: Description of a new species of the Neotropical cichlid genus Gymnogeophagus Miranda Ribeiro, 1918 (Teleostei: Cichliformes) from the Middle Paraná basin, Misiones, Argentina
Source: PLoS One. 2019 Feb 13;14(2):e0210166. doi: 10.1371/journal.pone.0210166 (PMC6374015; doi:10.1371/journal.pone.0210166)
Supplement: S3 Appendix — (PDF) [file pone.0210166.s003.pdf]

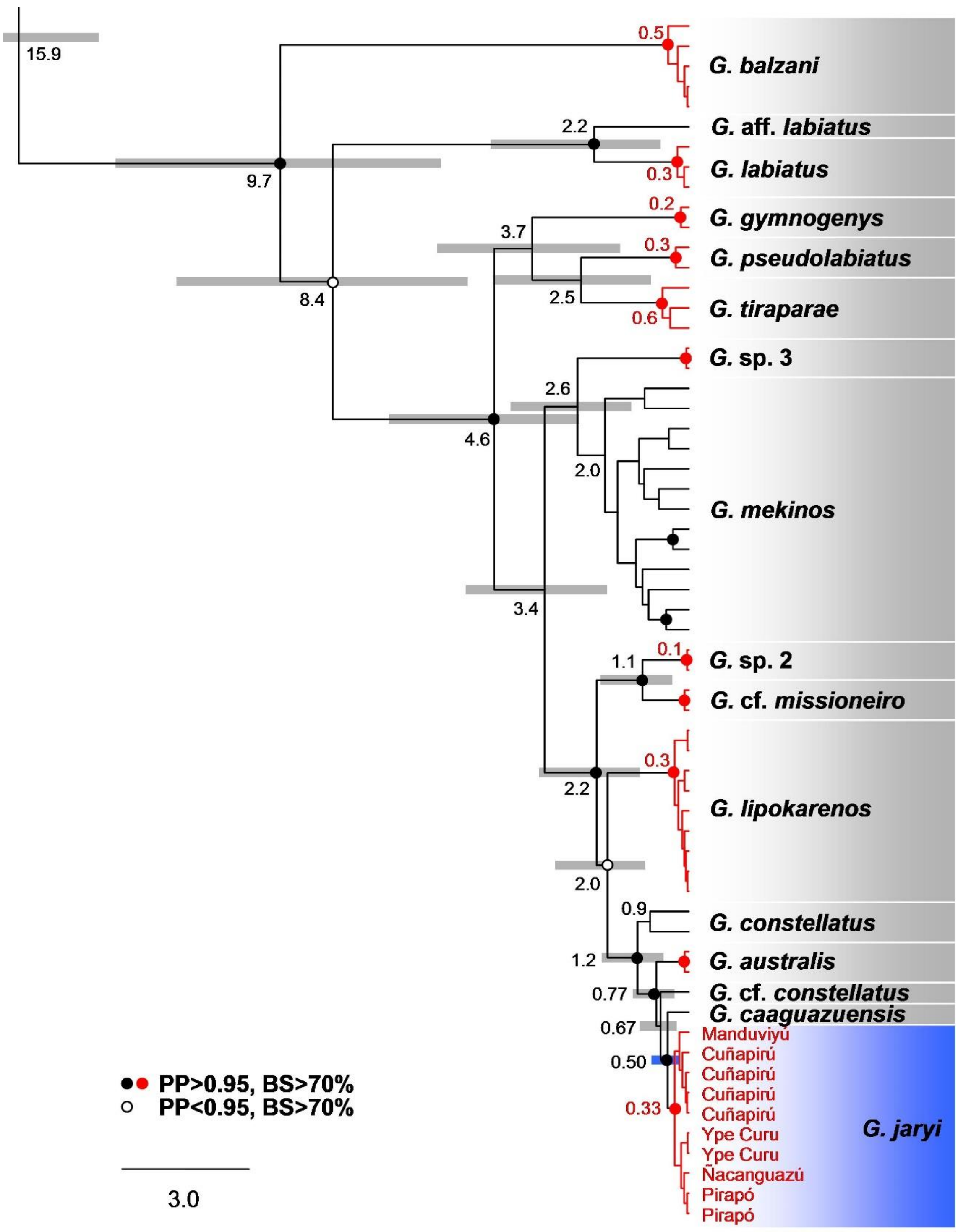

Phylogenetic relationships and molecular species delimitation within the *Gymnogeophagus gymnogenys* group based on the cytb marker from BEAST analysis. Rooting of the tree was done as in Říčan et al. (2018). Red branches in the tree are intraspecific based on species delimitation from bPTP analysis, black branches are

interspecific (results of the congruent but less conservative GMYC analysis are the same as in Říčan et al., 2018; not shown). Bars at nodes show confidence intervals of age estimates (95% HPD). Numbers at nodes show mean estimated divergences in Ma. Posterior probability (PP) of nodes from the Bayesian analysis and Bootstrap supports (BS) from a parsimony analysis in PAUP are shown at nodes.

References

Říčan, O., Říčanová, Š., Dragová, K., Piálek, L., Almirón, A., & Casciotta, J. 2018. Species diversity in Gymnogeophagus (Teleostei: Cichlidae) and comparative biogeography of cichlids in the Middle Paraná basin, an emerging hotspot of fish endemism. Hydrobiologia, 1-24.
